# Ultraviolet radiation‐induced degradation of dermal extracellular matrix and protection by green tea catechins: a randomized controlled trial

**DOI:** 10.1111/ced.15179

**Published:** 2022-05-18

**Authors:** Nisamanee Charoenchon, Lesley E. Rhodes, Anna Nicolaou, Gary Williamson, Rachel E. B. Watson, Mark D. Farrar

**Affiliations:** ^1^ Centre for Dermatology Research Division of Musculoskeletal and Dermatological Sciences School of Biological Sciences Faculty of Biology, Medicine and Health The University of Manchester, Manchester Academic Health Science Centre Manchester UK; ^2^ Photobiology Unit Dermatology Centre Salford Royal Hospital Manchester Academic Health Science Centre Manchester UK; ^3^ Laboratory for Lipidomics and Lipid Biology Division of Pharmacy and Optometry School of Health Sciences Faculty of Biology, Medicine and Health The University of Manchester, Manchester Academic Health Science Centre Manchester UK; ^4^ Department of Nutrition, Dietetics and Food School of Clinical Sciences at Monash Health Faculty of Medicine, Nursing and Health Sciences Monash University Melbourne VIC Australia

## Abstract

**Background:**

Loss and remodelling of the dermal extracellular matrix (ECM) are key features of photodamaged human skin. Green tea catechins (GTCs) have been explored for their anti‐inflammatory and chemopreventive properties, but data on the impact of GTCs on ultraviolet radiation (UVR)‐induced changes to the dermal ECM are lacking.

**Aim:**

To investigate the effect of an inflammatory dose of solar‐simulated UVR on human dermal ECM and potential for protection by GTCs in a double‐blind randomized controlled trial.

**Methods:**

In total, 50 healthy white (Fitzpatrick skin type I–II) adults aged 18–65 years were randomized to a combination of GTCs 540 mg plus vitamin C 50 mg or to placebo twice daily for 12 weeks. The impact of solar‐simulated UVR at 3 × minimal erythema dose on the dermal collagen and elastic fibre networks was assessed by histology and immunohistochemistry in all participants at baseline. The impact of GTC supplementation on UVR‐induced effects was compared between the groups post‐supplementation.

**Results:**

The area of papillary dermis covered by collagen and elastic fibres was significantly lower (*P* < 0.001) in UVR‐exposed skin than in unexposed skin. Significantly lower levels of fibrillin‐rich microfibrils (*P* = 0.02), fibulin‐2 (*P* < 0.001) and fibulin‐5 (*P* < 0.001) were seen in UVR‐exposed than unexposed skin, while procollagen‐1 deposition was significantly higher in UVR‐exposed skin (*P* = 0.01). Following GTC supplementation, the UVR‐induced change in fibulin‐5 was abrogated in the active group but not the placebo group, with no difference between the two groups for other components.

**Conclusions:**

Acute UVR induced significant changes in the human dermal collagen and elastic fibre networks, whereas oral GTCs conferred specific UVR protection to fibulin‐5. Future studies could explore the impact of GTCs on the effects of repeated suberythemal UVR exposure of human skin.

## Graphical abstract

Chronic exposure of human skin to ultraviolet radiation (UVR) in sunlight leads to loss and remodelling of dermal extracellular matrix components, acute UVR exposure may also contribute to photodamage. A single sunburn dose of solar‐simulated UVR caused significant reduction in the global collagen and elastic fibre networks, specifically fibrillin‐rich microfibrils, fibulin‐2 and fibulin‐5. Oral supplementation with green tea catechins abrogated the UVR‐induced reduction in fibulin‐5 with no significant effect on other dermal matrix parameters.

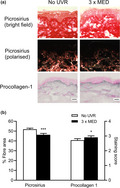

## Introduction

Dermal extracellular matrix (ECM) proteins include strength‐providing collagens and elastic fibre proteins, giving skin flexibility and recoil properties. The epidermis undergoes constant renewal whereas the dermis is relatively static. Dermal ECM components accrue damage over their life course, which negatively affects their structure and function and thus, the biomechanical properties of the skin.^1^, ^2^

Human skin is continually exposed to solar ultraviolet radiation (UVR), and repeated long‐term exposure to UVR causes skin photodamage, with commonly exposed sites such as the face and hands at greatest risk. Photoaged skin is characterized by a leathery, rough appearance with areas of hyperpigmentation and deep wrinkles.^3^ These visible features of photodamage are thought to result from direct and indirect effects of UVR on the proteins of the dermal ECM. One of the first pathological changes observed in photodamaged skin is loss of oxytalan fibres [fibrillin‐rich microfibrils (FRMs) and fibulin microfibrils] in the papillary dermis.^4^, ^5^ *In vitro*, UVB was demonstrated to alter fibrillin‐rich microfibril structure^6^ while *in vivo*, human pilot studies have shown that a single sunburn dose of solar‐simulated radiation (SSR) can reduce papillary dermal elastic fibres^7^ and procollagen‐1^8^ in photoprotected skin.

Photoprotection strategies largely involve topical sunscreens that absorb and scatter UVR, but sunscreen application by users is often inadequate.^9^ Furthermore, sunscreens are used infrequently during daily activity outside holiday time, potentially reducing protection from daily, suberythemal UVR exposures.^10^, ^11^ There is substantial interest in new approaches to complement sunscreens, particularly systemic photoprotection through diet. Green tea catechins (GTCs) have been demonstrated to protect against UVR‐induced inflammation and DNA damage in humans, although the data are conflicting.^12^, ^13^, ^14^, ^15^, ^16^

Data are lacking on the changes in dermal ECM following acute UVR exposure, and the impact of GTCs on these. Thus, our objectives were to: (i) examine changes in the dermal collagen and elastic fibre systems following SSR exposure, and (ii) examine the potential of oral GTC supplementation to protect against these changes, in a double‐blind, randomized controlled trial (RCT).

## Methods

### Participants

Healthy white adults aged 18–65 years with Fitzpatrick sun‐reactive skin types I–II were recruited by open advertising. Exclusions included a history of skin cancer or photosensitivity; sunbed use/sunbathing in the 3 months prior to the study; use of photoactive medication or nutritional supplements; consumption of > 2 cups of tea per day (which is any tea, i.e. green or black, but not herbal/fruit teas); or pregnancy/lactation.

### Study design

A double‐blind RCT was performed in the Photobiology Unit (Salford Royal Hospital, Greater Manchester, UK) between November 2010 and August 2011. The 50 enrolled participants were randomly assigned (1 : 1) to receive green tea extract plus vitamin C, or placebo (maltodextrin; no GTCs or vitamin C) for 12 weeks. Allocation was by block randomization (random blocks of 4–8, generated by MDF), coding was broken only after study completion.

Green tea supplements were provided as gelatine capsules, each containing green tea extract 450 mg (GTCs 180 mg).^14^ A separate set of capsules each contained vitamin C 25 mg to stabilize GTCs in the gut. Participants in the GTC group took three green tea and two vitamin C capsules twice daily with breakfast and evening meal; total daily dose of GTCs 1080 mg (equivalent to five cups of green tea) and vitamin C 100 mg; participants in the placebo group took maltodextrin in gelatine capsules that were identical in appearance to those containing green tea and vitamin C.^15^ Supplements were packaged in identical coded bottles then sequentially numbered to conceal allocation. Compliance was assessed by measurement of the urinary epigallocatechin glucuronide by liquid chromatography–tandem mass spectrometry.^17^

### Ultraviolet radiation exposure and skin biopsy

Exposure to UVR mimicking sunlight (290–400 nm; 5% UVB, 95% UVA) was performed using a solar‐simulator (Newport Spectra‐Physics Ltd, Didcot, Oxfordshire, UK). Minimal erythema dose (MED) was assessed at baseline, and challenges of 3 × MED were given before and after supplementation (Supplementary Data S1).

### Histology/immunohistochemistry

Biopsy sections were stained with Weigert resorcin fuchsin or picrosirius red to identify dermal elastic fibres and mature fibrillar collagens, respectively. Immunohistochemistry identified specific components of oxytalan fibres and newly synthesized procollagen (Supplementary Data S1). Staining was quantified as percentage of papillary dermal area from the dermoepidermal junction to a depth of 100 μm across the entire section, measured using ImageJ software (National Institutes of Health, Bethesda, MD, USA). Procollagen‐1 staining was assessed using a five‐point semi‐quantitative scale from 0 = no staining to 5 = maximal staining in the papillary dermis proximal to the dermoepidermal junction.

### Statistical analysis

The RCT was powered for UVR‐induced skin erythema outcomes as reported previously^15^ with dermal matrix parameters as secondary outcomes. To examine baseline effect of UVR on collagen and elastic fibre networks, paired t‐tests were used to compare dermal matrix parameters in unexposed and UVR‐exposed skin in all volunteers. The ability of GTCs to abrogate UVR‐induced changes in dermal matrix parameters was examined between groups post‐supplementation. Differences in UVR‐exposed skin parameters between groups were compared by ANCOVA with pre‐supplementation data as the covariate. Analyses were performed using SPSS (V20; IBM SPSS, Armonk, NY, USA). Statistical significance was accepted at *P* < 0.05.

## Results

### Participant flow and baseline characteristics

Participant flow and samples obtained are shown in Supplementary Fig. S1. Five participants (four active, one placebo) were noncompliant and excluded from analysis. The baseline characteristics of completing compliant participants providing all biopsy samples are shown in Supplementary Table S1.

### Modulation of dermal extracellular matrix by ultraviolet radiation

**Figure 1:**
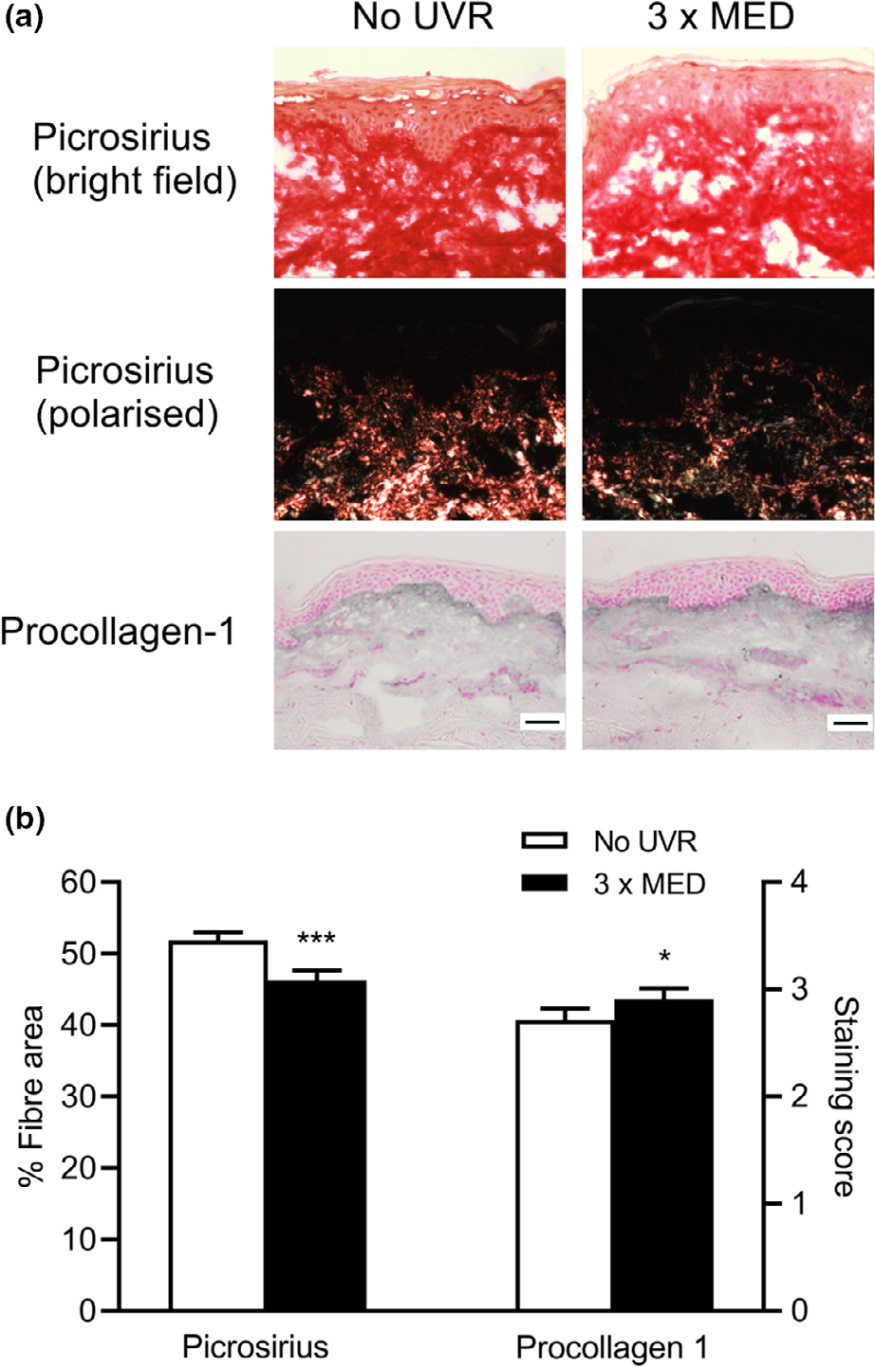
(a,b) Ultraviolet radiation (UVR)‐induced modulation of the collagen fibre network. (a) Representative images showing picrosirius red staining of collagen fibres (under bright‐field and polarized light microscopy) and immunohistochemistry of procollagen‐1 in the papillary dermis of UVR‐exposed and unexposed skin. Scale bar = 50 μm. (b) Mean ± SEM fibre area (picrosirius red) and staining score (procollagen‐1), *n* = 44; *P < 0.05, ***P < 0.001 compared with unexposed skin (paired *t*‐test). MED, minimal erythema dose. [Colour figure can be viewed at wileyonlinelibrary.com]

Picrosirius red staining of the mature fibrillar collagen network at baseline showed that the mean ± SD dermal area covered by collagen fibres was 46.3 ± 9.1% in skin exposed to 3 × MED UVR, which was significantly lower than in unexposed skin (51.8 ± 8.2%) (*P* < 0.001) (Fig. 1). Immunohistochemistry showed that UVR‐exposed skin had significantly higher deposition of procollagen‐1 (staining score 2.9 ± 0.7) compared with unexposed skin (2.7 ± 0.7) (*P* = 0.01) (Fig. 1).

**Figure 2:**
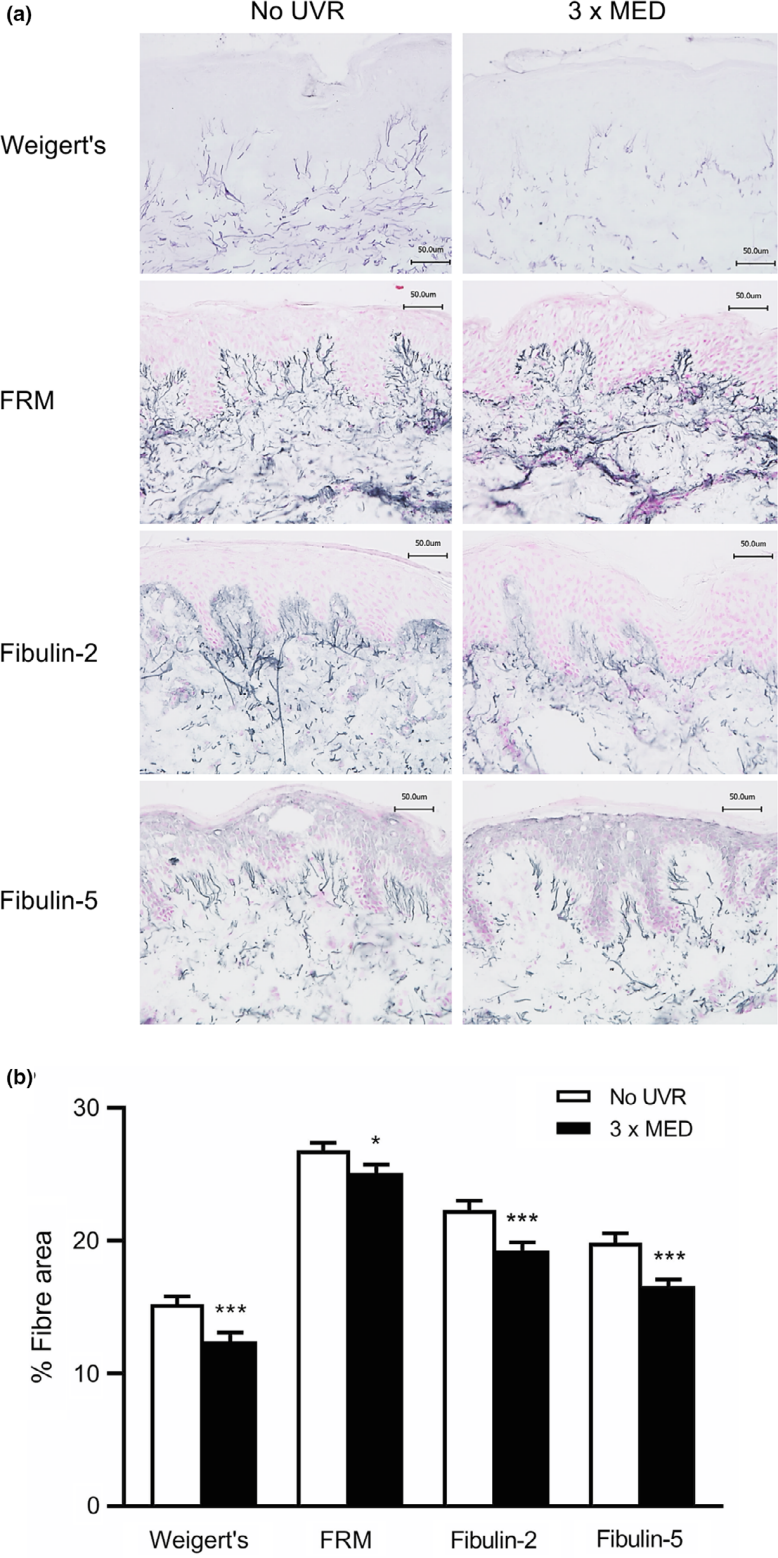
(a,b) Ultraviolet radiation (UVR)‐induced modulation of the elastic fibre network. (a) Representative images showing Weigert resorcin fuchsin staining of elastic fibres and immunohistochemistry of fibrillin‐rich microfibrils (FRMs), fibulin‐2 and fibulin‐5 in the papillary dermis of UVR‐exposed and unexposed skin. Scale bar = 50 μm. (b)  Mean ± SEM fibre area, *n* = 44; *P < 0.05, ***P < 0.001 compared with unexposed skin (paired *t*‐test). MED, minimal erythema dose. [Colour figure can be viewed at wileyonlinelibrary.com]

Similarly, Weigert staining of the dermal elastic fibre network in baseline skin showed that the mean ± SD dermal area covered by elastic fibres was 12.4 ± 4.3% in UVR‐exposed skin, significantly lower than in unexposed skin (15.2 ± 3.9%) (*P* < 0.001) (Fig. 2). Immunohistochemical analysis was performed to examine specific components of oxytalan fibres in the papillary dermis.

Significantly lower coverage of FRMs was observed in UVR‐exposed skin (25.1 ± 4.1%) compared with unexposed skin (26.8 ± 3.7%) (*P* = 0.02) (Fig. 2). Similarly, coverage of fibulin‐2 and fibulin‐5 was significantly lower in UVR‐exposed skin compared with unexposed skin. Mean coverage of fibulin‐2 was 19.3 ± 4.2% and 22.3 ± 4.5% in UVR‐exposed and unexposed skin, respectively (*P* < 0.001), while coverage of fibulin‐5 was 16.6 ± 3.4% and 19.9 ± 4.6%, respectively (*P* < 0.001) (Fig. 2).

### Impact of supplementation with green tea catechins on ultraviolet radiation‐induced modulation of dermal collagen fibres

**Figure 3:**
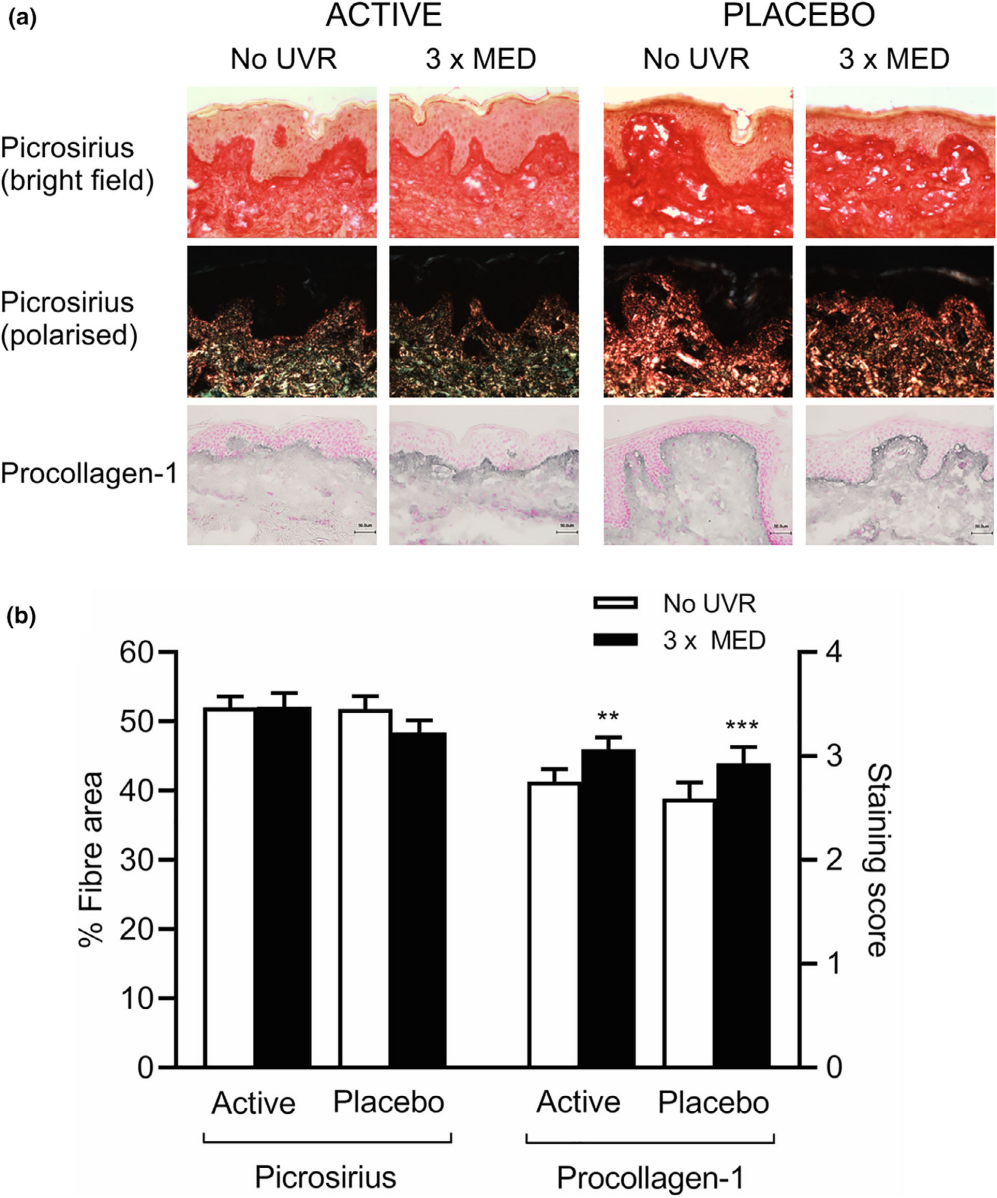
(a,b) Ultraviolet radiation (UVR)‐induced modulation of the collagen fibre network following 12‐week oral supplementation with green tea catechins (active: *n* = 20) or placebo (*n* = 24). (a) Representative images showing picrosirius red staining of collagen fibres (under bright‐field and polarized light microscopy) and immunohistochemistry of procollagen‐1 in the papillary dermis of UVR‐exposed and unexposed skin. Scale bar = 50 μm. (b) Mean ± SEM fibre area (picrosirius red) and staining score (procollagen‐1); ***P < 0.001 compared with unexposed skin (paired *t*‐test). MED, minimal erythema dose. [Colour figure can be viewed at wileyonlinelibrary.com]

Following 12 weeks of oral supplementation with GTCs or placebo, analysis of skin biopsy sections showed the mean collagen fibre coverage area was unchanged by UVR exposure in both the active group (unexposed: 52.0 ± 7.2%, UVR‐exposed: 52.1 ± 9.1%; *P* = 0.97) and placebo group (unexposed: 51.8 ± 9.0%, UVR‐exposed 48.4 ± 8.7%; *P* = 0.15) with no significant difference in UVR‐exposed skin between the groups (*P* = 0.11) (Fig. 3).

The UVR‐induced deposition of procollagen‐1 was maintained in participants in both the active and placebo groups (Fig. 3). Mean procollagen‐1 staining score was 3.1 ± 0.5 and 2.8 ± 0.5 in UVR‐exposed and unexposed skin respectively (*P* < 0.01) in the active group, and 2.9 ± 0.8 and 2.6 ± 0.8 respectively (*P* < 0.001) in the placebo group. There was no significant difference in procollagen‐1 staining in UVR‐exposed skin between the groups post‐supplementation (*P* = 0.81).

### Impact of supplementation with green tea catechins on ultraviolet radiation‐induced modulation of dermal elastic fibres

**Figure 4:**
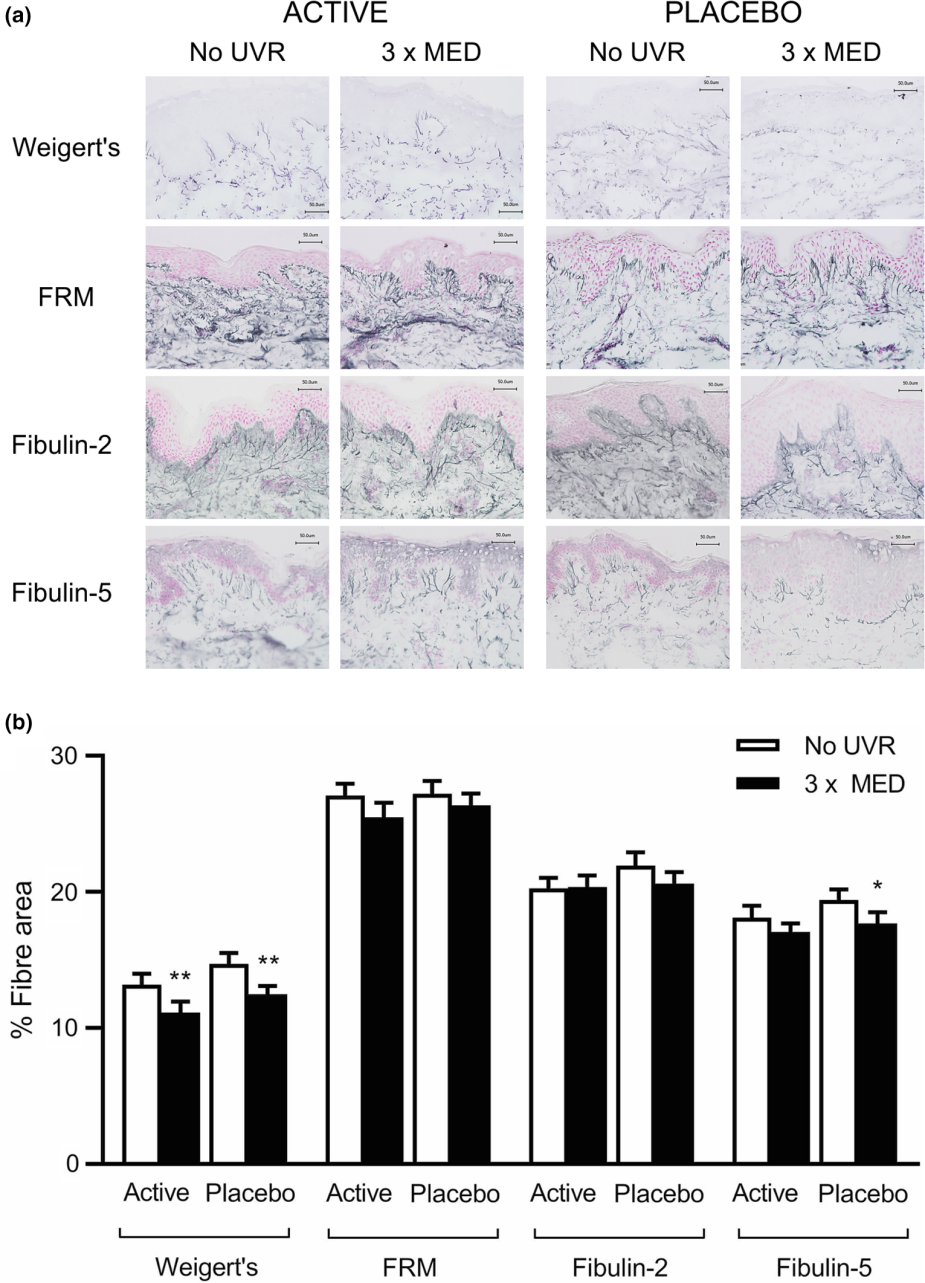
(a,b) Ultraviolet radiation (UVR)‐induced modulation of the elastic fibre network following 12‐week oral supplementation with green tea catechins (active; *n* = 20) or placebo (*n* = 24). (a) Representative images showing Weigert resorcin fuchsin staining of elastic fibres and immunohistochemistry of fibrillin‐rich microfibrils (FRM), fibulin‐2 and fibulin‐5 in the papillary dermis of UVR‐exposed and unexposed skin. Scale bar = 50 μm. (b) Mean ± SEM fibre area; *P < 0.05, **P < 0.01 compared with unexposed skin (paired *t*‐test). MED, minimal erythema dose. [Colour figure can be viewed at wileyonlinelibrary.com]

Analysis of skin biopsies following supplementation showed overall elastic fibre area coverage to be lower in UVR‐exposed skin compared with unexposed skin in both the active group (11.2 ± 3.5% and 13.2 ± 3.5% respectively; *P* < 0.01) and placebo group (12.5 ± 3.0% and 14.7 ± 3.9% respectively; *P* < 0.01) with no significant difference in UVR‐exposed skin between groups (*P* = 0.55) (Fig. 4).

Assessment of specific fibre components showed fibrillin‐1 and fibulin‐2 coverage to be unchanged by UVR in both the active and placebo groups (Fig. 4). Mean FRM coverage in UVR‐exposed and unexposed skin was 25.5 ± 4.9% and 27.1 ± 3.8%, respectively, in the active group (*P* = 0.14) and 26.4 ± 4.3% and 27.2 ± 4.6%, respectively, in the placebo group (*P* = 0.45) with no difference in UVR‐exposed skin between groups (*P* = 0.28). Mean fibulin‐2 coverage in UVR‐exposed and unexposed skin was 20.4 ± 3.7% and 20.3 ± 3.5%, respectively, in the active group (*P* = 0.90), and 20.6 ± 4.1% and 21.9 ± 4.8%, respectively, in the placebo group (*P* = 0.06) with no difference in UVR‐exposed skin between groups (*P* = 0.82). Notably, in the placebo group, coverage of fibulin‐5 in UVR‐exposed skin remained significantly lower than coverage in unexposed skin post‐supplementation (17.7 ± 4.1% and 19.4 ± 3.8%, respectively; *P* = 0.01) but not in the active group, in which no significant difference between UVR‐exposed and unexposed skin was seen (17.1 ± 2.7% and 18.1 ± 4.0%, respectively; *P* = 0.30). There was no significant difference in fibulin‐5 coverage in UVR‐exposed skin between groups (*P* = 0.65).

## Discussion

Human skin is regularly exposed to UVR in sunlight, leading to degradation and remodelling of the dermal ECM, which is a key feature of photodamage. Dietary interventions have gained substantial interest as a photoprotective measure to mitigate the damaging effects of solar UVR, with green tea demonstrated to have benefit in animal models and a small number of human studies,^12^, ^14^, ^18^ but its impact on photodamage remained unexplored. We examined modulation of dermal ECM components following a single, reproducible sunburn dose of solar‐simulated UVR, and assessed the potential of oral GTCs with vitamin C to provide photoprotection through a double‐blind RCT.

In photoprotected skin, the levels of mature collagen and elastic fibres in the papillary dermis were significantly lower in UVR‐exposed skin than in unexposed skin. Immunostaining revealed the area covered by FRM, fibulin‐2 and fibulin‐5 to be significantly lower in UVR‐exposed skin than unexposed skin, whereas deposition of procollagen‐1 was greater in UVR‐exposed skin. The median UVR dose received by our participants was 84 mJ/cm^2^ (8.4 standard erythema doses), equivalent to 75–100 min of UK sunlight exposure around midday in June, depending on latitude.^19^ Oral GTCs did not modulate the impact of UVR exposure on global collagen fibre or elastic fibre networks, procollagen‐1 deposition, or FRM or fibulin‐2 area coverage. However, compared with placebo, GTCs reduced UVR‐induced changes in fibulin‐5, a key elastic fibre component involved in regulation of fibre assembly in human skin fibroblasts.^20^

*In vivo* studies on the effects of acute UVR challenge on human dermal ECM are few, and most of them used UVA or UVB sources rather than SSR. We previously reported reorganization of the global elastic fibre network and specifically fibulin‐5 in response to acute SSR challenge in a pilot human study of six participants,^7^ and the current study now demonstrates novel changes in FRMs, fibulin‐2 and the global mature collagen fibre network. In a previous study, modulation of collagen deposition was reported in mice following repeated suberythemal UVA or UVB exposure,^21^ but to our knowledge, the effect of an acute dose of SSR on the dermal collagen network in human skin has not been reported, and our data further support public health advice to avoid sunburn. The impact of ethnicity and skin pigmentation on this acute photodamage requires further investigation.

The mechanisms underlying our observations may involve both direct and indirect processes. Elastic fibre network proteins contain a relatively high proportion of UVR‐absorbing amino acids, which are hypothesized to mediate direct photodegradation.^6^ These amino acids are also oxidation‐sensitive and therefore susceptible to the action of UVR‐generated reactive oxygen species. Oxidatively modified proteins accumulate in human skin following repeated SSR exposure, which can result in aggregation, fragmentation and structural changes.^22^ Degradation and remodelling of ECM through the action of matrix metalloproteinases (MMPs) and other enzymes upregulated by UVR may also be involved.^23^ Our finding of higher deposition of procollagen‐1 following UVR exposure is intriguing, and suggests that fibroblast collagen synthesis may be induced to counteract the degradative effects of UVR.

Our double‐blind RCT found that oral supplementation with GTCs specifically protected fibulin‐5 from UVR‐induced modulation compared with placebo. Similar findings were noted for fibulin‐2, with the significant UVR‐induced reduction in dermal coverage abolished in the active group, whereas the reduction in the placebo group was close to reaching significance (*P* = 0.06). We previously showed that oral GTCs were bioavailable in human skin with interindividual variability in the catechin and metabolite types detected,^24^ but the mechanisms by which they could selectively protect ECM components are unclear. Many GTCs are antioxidants, and thus may protect those components more vulnerable to oxidative damage. Elastic fibres are susceptible to degradation by MMPs, and there is evidence that epigallocatechin gallate, the major GTC, can attenuate the activity of certain MMPs.^25^ However, MMPs have broad activity and we did not observe an effect of GTCs on other specific ECM components or on the overall ECM networks.

Vitamin C, included in the active supplement to stabilize GTCs, is vital for collagen synthesis. The impact of vitamin C supplementation on tissue healing following injury has been investigated but with little evidence of an effect in humans,^26^ and there is no evidence of vitamin C enhancing collagen production in the skin of healthy, vitamin C‐replete humans. Thus, the addition of vitamin C with GTCs is unlikely to have confounded findings in our study.

The limitations of this study include that it was powered for erythema outcomes rather than dermal photodamage parameters, and used a single, relatively high dose of UVR (3 × MED) with outcomes assessed at a single time point (24 h). Thus, our study may not have had sufficient power or an optimal dose/time point to detect differences in some outcomes, and further studies are warranted.

## Conclusion

A sunburn dose of SSR induced significant changes in components of the dermal collagen and elastic fibre networks in human skin. Notably, 12‐week supplementation with GTCs and vitamin C conferred specific protection of fibulin‐5, and potentially fibulin‐2, against these UVR‐induced effects. Further studies should examine the mechanisms underlying this protection, and explore the effects of repeated suberythemal UVR exposure on photoageing pathology, and the effects of GTCs on this, under conditions reflecting an individual's daily sunlight exposure scenario.

**What's already known about this topic?**

• Chronic exposure of human skin to UVR in sunlight leads to loss and remodelling of dermal ECM components.
• Acute UVR exposure may also contribute to photodamage.
• Limited studies have explored the impact of GTCs on the clinical features of skin photodamage in humans.

**What does this study add?**

• A single sunburn dose of solar‐simulated UVR caused a significant reduction in the global collagen and elastic fibre networks.
• The area of papillary dermis covered by fibrillin‐rich microfibrils, fibulin‐2 and fibulin‐5 was significantly reduced in UVR‐exposed skin.
• Oral supplementation with GTCs abrogated the UVR‐induced reduction in fibulin‐5 with no significant effect on other dermal matrix parameters.

## Conflict of interest

The authors declare that they have no conflicts of interest.

## Funding

This work was funded by grants BB/G005575/1 (LER), BB/G005540/1 (AN) and BB/G005559/1 (GW) from the Biotechnology and Biological Sciences Research Council, Diet and Health Research Industry Club (BBSRC DRINC) and supported by the NIHR Manchester Biomedical Research Centre (LER, AN, REBW, MDF). NC was on a studentship funded by the Royal Government of Thailand Development and Promotion of Science and Technology Talents Project.

## Ethics statement

The study was approved by the North Manchester Research Ethics Committee (08/H1006/79) and the study adhered to the Declaration of Helsinki principles. Participants provided written informed consent to participation and publication of results.

## Clinical trials number

This study is listed in the ClinicalTrials.gov registry (www.clinicaltrials.gov) with (NCT01032031).

## Data availability

The data that support the findings of this study are available from the corresponding author upon reasonable request.

## Supporting information


**Supplementary Data S1.** Supplementary Methods.
**Supplementary Figure S1.** Participant flow and skin biopsy samples obtained. Of the participants randomized to GTCs, three refused skin biopsies at baseline and post‐supplementation and two of these were noncompliant with the intervention. A further two participants in the green tea catechins (GTCs) group were noncompliant thus 20 compliant participants provided biopsies. Of those randomized to placebo, 1 patient was noncompliant, thus 24 compliant participants provided biopsies.
**Supplementary Table S1.** Baseline characteristics of completing compliant subjects providing biopsies.
