# Supplementary material for: Ultraviolet radiation‐induced degradation of dermal extracellular matrix and protection by green tea catechins: a randomized controlled trial
Source: Clin Exp Dermatol. 2022 May 18;47(7):1314–23. doi: 10.1111/ced.15179 (PMC9320810; doi:10.1111/ced.15179)
Supplement: Supplementary file 1 — Supplementary Data S1. Supplementary Methods. Supplementary Figure S1. Participant flow and skin biopsy samples obtained. Of the participants randomized to GTCs, three refused skin biopsies at baseline and post‐supplementation and two of these were noncompliant with the intervention. A further two participants in the green tea catechins (GTCs) group were noncompliant thus 20 compliant participants provided biopsies. Of those randomized to placebo, 1 patient was noncompliant, thus 24 compliant participants provided biopsies. Supplementary Table S1. Baseline characteristics of completing compliant subjects providing biopsies. [file CED-47-1314-s001.docx]

**Supplementary Methods**

**Histology and immunohistochemistry**

To identify the elastic fibre network, frozen biopsy sections (7µm) were fixed in 4% (w/v) paraformaldehyde in PBS prior to immersion in Weigert’s haematoxylin (Millipore, Darmstadt, Germany). Sections were then washed with industrial methylated spirits (IMS), rinsed in distilled water and stained with Weigert’s resorcin fuchsin (Clin-Tech Ltd., Guilford, UK). To identify the mature fibrillar collagen network, sections were stained with picrosirius red. Sections were fixed in 90% IMS then briefly immersed in 0.1% (w/v) sirius red F3BA in saturated aqueous picric acid. Excess stain was removed by rinsing with 0.1% (v/v) acetic acid and deionised water. Stained sections were dehydrated through serial alcohols (70-100% IMS), cleared in xylene and permanently mounted (DePex, Fisher Chemical, Loughborough, UK).

For immunohistochemistry, sections were fixed in 4% (w/v) paraformaldehyde in PBS prior to membrane permeabilisation with 0.5% (v/v) Triton X100. Endogenous peroxidase activity was quenched using H_2_O_2_ in methanol, sections were blocked with normal serum with bovine serum albumin, then incubated overnight at 4°C with primary antibody: monoclonal anti-FRM (clone 11C1.3, 1:1000; NeoMarkers; Fremont, CA USA); polyclonal anti-fibulin-2 (HPA001934, 1:1000; Atlas Antibodies AB, Stockholm, Sweden); polyclonal anti-fibulin-5 (HPA000848, 1:180; Atlas Antibodies AB, Stockholm, Sweden); monoclonal anti-procollagen-1 (clone M58, 1:200; Millipore, Darmstadt, Germany). Binding was visualised using biotinylated secondary antibodies (Vector *Elite* ABC; Vector Labs, Burlingame, USA) and Vector SG^®^ chromogen (Vector Labs). Nuclei were counterstained with Nuclear Fast Red (Vector Labs), serially dehydrated, cleared and permanently mounted.

For each parameter, three sections were analysed per biopsy with sections randomised and blinded prior to image capture. Sections stained with picrosirius red were imaged by polarisation and bright-field microscopy (Leitz DMRB, Leica Microsystems, Buffalo Grove, USA). All other sections were imaged under bright-field using a Biozero-800 all-in-one microscope (Keyence, Osaka, Japan).

**UVR exposures**

Irradiance was measured prior to each exposure using a calibrated radiometer (IL730A; International Light, Peabody, USA) to ensure dose consistency. Minimal erythema dose (MED) was assessed at baseline through application of a geometric 10 dose series of solar-simulated UVR (erythemally weighted, 7-80mJ/cm^2^) to upper buttock skin. The MED was the lowest dose producing just perceptible erythema 24 h after irradiation. Prior to supplementation, a UVR challenge of 3MED was given to photoprotected upper buttock to initiate ECM remodelling. At 24h post-exposure, punch biopsies (5mm) were taken from UVR-exposed skin and from unexposed skin on the contralateral buttock. Skin samples were snap frozen and stored at -80°C until analysis. The UVR challenge and biopsies were repeated following the 12-week supplementation period.

**Supplementary Table**

**Table S1.** Baseline characteristics of completing compliant subjects providing biopsies

| Characteristic | Active (*n* = 20) | Placebo (*n* = 24) |
| --- | --- | --- |
| Age*^1^* (y) | 35.5 ± 14.8 | 34.3 ± 11.0 |
| Sex (M/F), *n* | 5/15 | 7/17 |
| BMI*^1^* (kg/m^2^) | 27.3 ± 5.5 | 25.5 ± 3.9 |
| Skin type (I/II), *n* | 2/18 | 1/23 |
| MED*^2^* (mJ/cm^2^) | 28 (16-48) | 28 (7-48) |

*^1^*Mean ± SD

*^2^*Median (range); MED, minimal erythema dose

**Supplementary Figure**


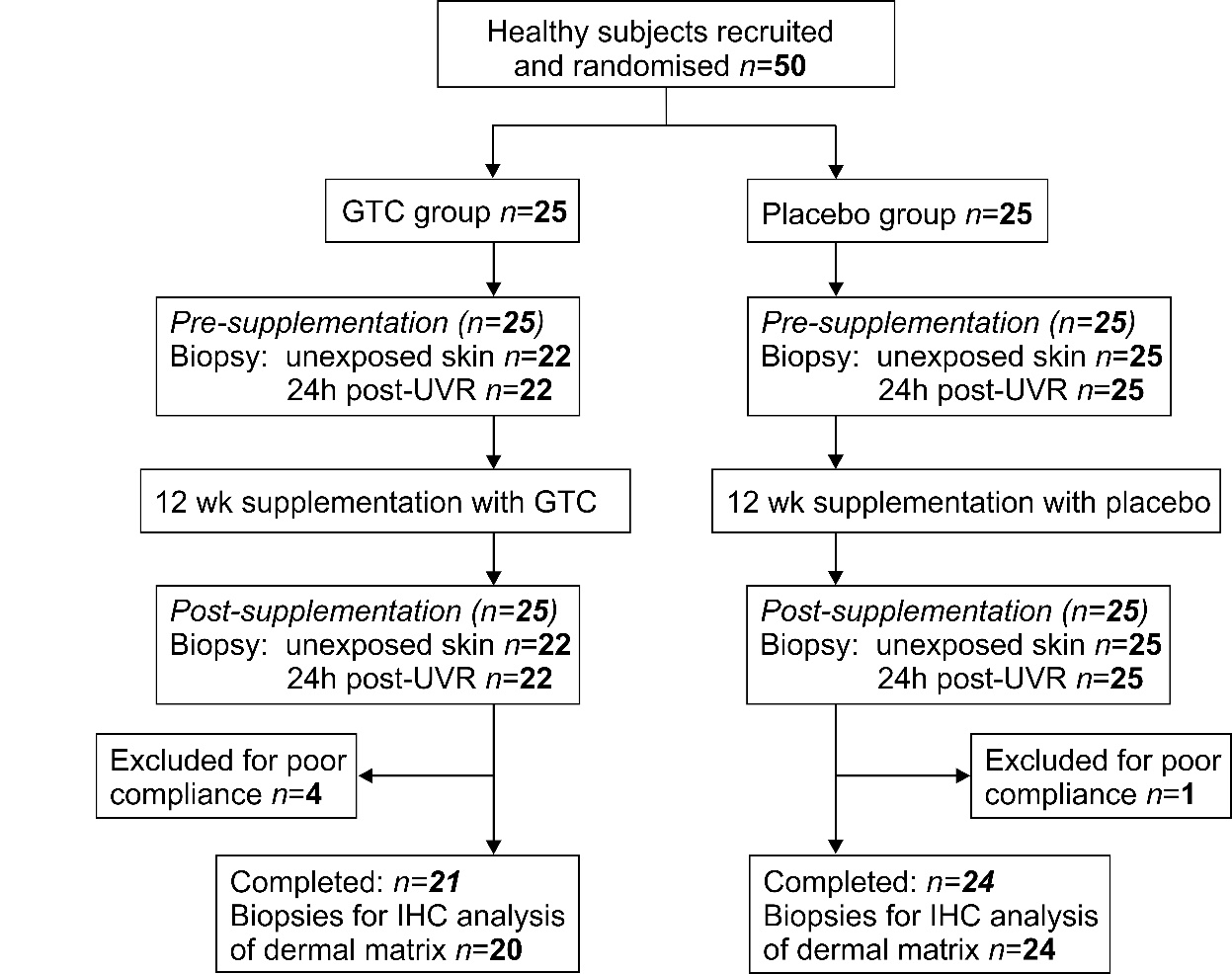


**Figure S1.** Participant flow and skin biopsy samples obtained. Of the participants randomised to GTC, n=3 refused skin biopsies at baseline and post-supplementation and n=2 of these were non-compliant with the intervention. A further n=2 participants in the GTC group were non-compliant thus n=20 compliant participants provided biopsies. Of those randomised to placebo, n=1 was non-compliant thus n=24 compliant participants provided biopsies. GTC, green tea catechins.
